# Prognostic impact of concurrent *MYC* and *BCL6* rearrangements and expression in *de novo* diffuse large B-cell lymphoma

**DOI:** 10.18632/oncotarget.6262

**Published:** 2015-11-12

**Authors:** Qing Ye, Zijun Y. Xu-Monette, Alexandar Tzankov, Lijuan Deng, Xiaoxiao Wang, Ganiraju C. Manyam, Carlo Visco, Santiago Montes-Moreno, Li Zhang, Karen Dybkær, April Chiu, Attilio Orazi, Youli Zu, Govind Bhagat, Kristy L. Richards, Eric D. Hsi, William W.L. Choi, J. Han van Krieken, Jooryung Huh, Maurilio Ponzoni, Andrés J.M. Ferreri, Ben M. Parsons, Michael B. Møller, Miguel A. Piris, Jane N. Winter, L. Jeffrey Medeiros, Shimin Hu, Ken H. Young

**Affiliations:** ^1^ Department of Hematopathology, The University of Texas MD Anderson Cancer Center, Houston, Texas, USA; ^2^ University Hospital, Basel, Switzerland; ^3^ Department of Computational Biology and Bioinformatics, The University of Texas MD Anderson Cancer Center, Houston, Texas, USA; ^4^ San Bortolo Hospital, Vicenza, Italy; ^5^ Hospital Universitario Marques de Valdecilla, Santander, Spain; ^6^ Aalborg University Hospital, Aalborg, Denmark; ^7^ Memorial Sloan-Kettering Cancer Center, New York, New York, USA; ^8^ Weill Medical College of Cornell University, New York, New York, USA; ^9^ Houston Methodist Hospital, Houston, Texas, USA; ^10^ Columbia University Medical Center and New York Presbyterian Hospital, New York, New York, USA; ^11^ University of North Carolina School of Medicine, Chapel Hill, North Carolina, USA; ^12^ Cleveland Clinic, Cleveland, Ohio, USA; ^13^ University of Hong Kong Li Ka Shing Faculty of Medicine, Hong Kong, China; ^14^ Radboud University Nijmegen Medical Centre, Nijmegen, The Netherlands; ^15^ Asan Medical Center, Ulsan University College of Medicine, Seoul, Korea; ^16^ San Raffaele H. Scientific Institute, Milan, Italy; ^17^ Gundersen Lutheran Health System, La Crosse, Wisconsin, USA; ^18^ Odense University Hospital, Odense, Denmark; ^19^ Feinberg School of Medicine, Northwestern University, Chicago, Illinois, USA; ^20^ The University of Texas School of Medicine, Graduate School of Biomedical Sciences, Houston, Texas, USA

**Keywords:** diffuse large B-cell lymphoma, double-hit, MYC, BCL6, BCL2

## Abstract

Double-hit B-cell lymphoma is a common designation for a group of tumors characterized by concurrent translocations of *MYC* and *BCL2*, *BCL6*, or other genes. The prognosis of concurrent *MYC* and *BCL6* translocations is not well known. In this study, we assessed rearrangements and expression of *MYC*, *BCL2* and *BCL6* in 898 patients with *de novo* diffuse large B-cell lymphoma treated with standard chemotherapy (cyclophosphamide, doxorubicin, vincristine, and prednisone plus rituximab). Neither *BCL6* translocation alone (more frequent in activated B-cell like diffuse large B-cell lymphoma) nor in combination with *MYC* translocation (observed in 2.0% of diffuse large B-cell lymphoma) predicted poorer survival in diffuse large B-cell lymphoma patients. Diffuse large B-cell lymphoma patients with MYC/BCL6 co-expression did have significantly poorer survival, however, MYC/BCL6 co-expression had no effect on prognosis in the absence of MYC/BCL2 co-expression, and had no additive impact in MYC^+^/BCL2^+^ cases. The isolated MYC^+^/BCL6^+^/BCL2^−^ subset, more frequent in germinal center B-cell like diffuse large B-cell lymphoma, had significantly better survival compared with the isolated MYC^+^/BCL2^+^/BCL6^−^ subset (more frequent in activated B-cell like diffuse large B-cell lymphoma). In summary, diffuse large B-cell lymphoma patients with either *MYC/BCL6* rearrangements or MYC/BCL6 co-expression did not always have poorer prognosis; MYC expression levels should be evaluated simultaneously; and double-hit B-cell lymphoma needs to be refined based on the specific genetic abnormalities present in these tumors.

## INTRODUCTION

Diffuse large B-cell lymphoma (DLBCL) is the most common type of non-Hodgkin lymphoma and has heterogeneous biologic features. Chromosomal rearrangements, the biologic and diagnostic hallmarks of some other types of B-cell lymphoma, also occur in DLBCL. The most common chromosomal rearrangements in DLBCL are those involving chromosomal gene loci 8q24/*MYC,* 18q21/*BCL2*, and 3q27/*BCL6* [1, 2]. *MYC* rearrangement, a disease-initiating event in Burkitt lymphoma (BL), can be observed in approximately 10% of *de novo* DLBCL and correlates with a poorer outcome [3–7]. However, *MYC* rearrangement alone may not explain the poor prognosis of patients with DLBCL that carry *MYC* rearrangement plus another chromosomal rearrangement. The designation double-hit lymphoma (DHL) has been used for a B-cell lymphoma carrying a *MYC*/8q24 rearrangement in combination with a rearrangement involving either *BCL2*, *BCL6*, or rarely other known oncogenes [2, 8, 9].

By far, the most common and well-studied type of DHL is characterized by concurrent *MYC* and *BCL2* rearrangements (*MYC/BCL2* DHL), occurring in about 5% of all cases of DLBCL [10, 11]. As key regulators of cell proliferation and apoptosis, respectively, *MYC* and *BCL2* may act synergistically to drive the pathogenesis of *MYC/BCL2* DHL [12]. Clinically, patients with *MYC/BCL2* DHL often exhibit adverse prognostic factors, such as high serum lactate dehydrogenase (LDH) level, advanced stage of disease, extranodal involvement, and a high proliferation index with a median value of 90%. There is a general consensus that *MYC/BCL2* DHL represents a treatment-refractory subgroup with a median survival of approximately 8 months [13–20]. Despite the dismal outcome of patients with *MYC/BCL2* DHL, almost all of these tumors arise within the germinal center B cell-like (GCB) subtype, a generally favorable prognostic subtype, illustrating a discordance between clinical behavior and cell of origin (COO) subtypes [1, 15, 17, 21].

As an extension of the concept of *MYC/BCL2* DHL, the concept of “double protein lymphoma (DPL)” has been developed in recent years referring to DLBCL with coexpression of MYC and BCL2 detected by immunohistochemistry (IHC), regardless of activation mechanisms. MYC/BCL2 DPL is more common than *MYC/BCL2* DHL and accounts for 18-44% of DLBCL cases and might result from gene amplification, transcriptional dysregulation or both [11, 22–26]. A series of studies have shown that patients with MYC/BCL2 DPL have a significantly poorer outcome than patients who express only one or neither protein, with a 5-year progression-free survival (PFS) of 25% following R-CHOP treatment [11, 22]. Interestingly, unlike *MYC/BCL2* DHL which is mainly observed in the GCB subtype, MYC/BCL2 DPL is more common in the activated-B cell-like (ABC) subtype and may largely contribute to inferior survival via NF-κB pathway activation [23].

According to the concept of DHL used currently, another type of DHL is *MYC/BCL6* DHL with concurrent *MYC* and *BCL6* rearrangements [1, 2]. However, there are far less data available for *MYC/BCL6* DHL, in part because of its rarity. BCL6 is a transcriptional suppressor required for germinal center formation with numerous transcriptional targets, including the cell cycle regulator *CCND2* and *MYC*, which explains downregulation of *MYC* in normal germinal center B-cells [27–29]. Studies by others have suggested that BCL6 expression is associated with better survival of DLBCL patients [24, 30]. The frequency and the prognostic impact of concurrent *MYC*/*BCL6* rearrangements and MYC/BCL6 protein coexpression in DLBCL remain unclear.

In this study, we assessed the frequency, clinicopathologic features, and the prognostic impact of concurrent *MYC/BCL6* rearrangements or MYC/BCL6 coexpression in a large cohort of *de novo* DLBCL patients treated with R-CHOP, in comparison to *MYC/BCL2* rearrangements and MYC/BCL2 coexpression. The study evaluated the role of each genetic translocation separately and in combinations, providing reliable conclusion and practical recommendations for diagnostic workup and prognostic prediction.

## RESULTS

### Overall frequency and distribution

The median age of the study population was 64 years (range, 16-95). The median follow-up time was 58.9 months (range, 1-187 months). Among the 898 cases, 469 (52%) were GCB and 429 (48%) were determined to be GCB and ABC subtype, respectively. The complete response rate to R-CHOP therapy was 75%. As shown in Table 1, rearrangements of *MYC*, *BCL2*, and *BCL6* were detected in 71 (11.8%) of 600, 94 (13.6%) of 690, and 145 (23.1%) of 628 cases, respectively. *MYC* and *BCL2* rearrangements were detected predominantly in GCB subtype (*P* = 0.0005 and *P* < 0.0001, respectively) whereas *BCL6* rearrangement was more frequently observed in the ABC subtype (*P* = 0.0002). *MYC/BCL2*, *MYC/BCL6*, and *BCL2/BCL6* concurrent rearrangements were identified in 20 (2.8%), 14 (2%), and 21 (2.9%) patients, respectively. Both *MYC/BCL2* and *BCL2/BCL6* concurrent rearrangements were observed mostly in the GCB subtype (*MYC+/BCL2+*: *P* < 0.0001; *BCL2+/BCL6+*: *P* = 0.0045) whereas *MYC/BCL6* concurrent rearrangements were observed in two COO subtypes (9 GCB, 5 ABC) without significantly difference in frequency (*P* = 0.37).

**Table 1:** Frequencies of *MYC*, *BCL2* and *BCL6* gene translocations and protein overexpression, and multivariate survival analysis

	Overall	GCB	ABC	GCB *vs* ABC	**OS**		**PFS**	
	n/n (%)	n/n (%)	n/n (%)	*P* value	HR (95% CI)	*P* value	HR (95% CI)	*P* value
*MYC* translocation	71/600 (11.8%)	**51/314 (16.2%)**	20/284 (7.0%)	**.0005**	1.36 (0.83-2.23)	.22	1.26 (0.79-2.01)	.34
*BCL2* translocation	94/690 (13.6%)	**85/360 (23.6%)**	9/328 (2.7%)	**<.0001**	1.39 (0.86-2.24)	.17	1.34 (0.86-2.09)	.20
*BCL6* translocation	145/628 (23.1%)	59/338 (17.5%)	**86/286 (30.1%)**	**.0002**	1.06 (0.70-1.61)	.77	1.13 (0.77-1.66)	.53
MYC^+^ expression	249/825 (30.2%)	121/430 (28.1%)	127/390 (32.6%)	.17	1.89 (1.26-2.84)	**.002**	1.85 (1.26-2.71)	**.002**
BCL2^+^ expression	439/849 (51.7%)	194/439 (44.2%)	**245/406 (60.3%)**	**<.0001**	1.67 (1.14-2.46)	**.009**	1.70 (1.19-2.42)	**.004**
BCL6^+^ expression	555/887 (62.6%)	**350/462 (75.8%)**	204/424 (48.1%)	**<.0001**	0.67 (0.45-1.00)	**.048**	0.47 (0.40-0.89)	**.016**
*MYC*+/*BCL2*+ double-hit	20/710 (2.8%)	**19/359 (5.3%)**	1/349 (0.3%)	**<.0001**	3.38 (1.75-6.52)	**<.0001**	3.04 (1.63-5.67)	**<.0001**
*MYC*+/*BCL6*+ concurrent translocation	14/683 (2.0%)	9/356 (2.5%)	5/323 (1.5%)	.37	0.67 (0.23-1.97)	.47	0.85 (0.33-2.21)	.74
*BCL2*+/*BCL6*+ concurrent translocation	21/718 (2.9%)	**18/371 (4.9%)**	3/341 (0.9%)	**.0045**	0.47 (0.16-1.39)	.14	0.52 (0.20-1.33)	.17
MYC^+^/BCL2^+^ double-positive	146/831 (17.6%)	62/434 (14.3%)	**84/390 (21.5%)**	**.0079**	2.54 (1.65-3.94)	**<.0001**	2.92 (1.91-4.47)	**<.0001**
MYC^+^/BCL6^+^ co-expression	178/821 (21.7%)	97/434 (22.4%)	81/413 (19.6%)	.35	1.02 (0.65-1.58)	.94	0.85 (0.56-1.30)	.46
BCL2^+^/BCL6^+^ co-expression	282/845 (33.4%)	151/443 (34.1%)	131/415 (31.6%)	.47	1.01 (0.68-1.49)	.97	0.90 (0.62-1.30)	.58
MYC^+^/BCL2^+^/BCL6^−^	43/871 (4.9%)	13/460 (2.8%)	**30/403 (7.4%)**	**.0019**	1.60 (1.01-2.54)	**.046**	2.16 (1.39-3.34)	**.001**
MYC^+^/BCL6^+^/BCL2^−^	75/850 (8.8%)	**49/434 (11.3%)**	26/412 (6.3%)	**.011**	0.87 (0.54-1.40)	.56	0.86 (0.58-1.34)	.51
MYC^+^/BCL2^+^/BCL6^+^	101/829 (12.2%)	47/432 (10.9%)	54/390 (13.8%)	.20	2.57 (1.86-3.56)	**<.0001**	2.41 (1.77-3.28)	**<.0001**
(triple-positive)								

**Abbreviations**: OS, overall survival; PFS, progression-free survival; HR, hazard ratio; CI, confidence interval.

**Note**: (1) Not all the patients had experiment results available for all three genes (*MYC*/*BCL2*/*BCL6*) or protein mostly due to tissue exhaustion. Total patient numbers for particular biomarkers are listed. For defined combination biomarkers (double-hit/concurrent translocation or double positive/co-expression), total case numbers are more than those for single markers because some cases had only one gene/protein (as a component of the defined biomarker combinations) data available and the results were negative. For example, cases known to have no *MYC* translocation but without *BCL2* translocation status known are included as non *MYC*+/*BCL2*+ double-hit cases during frequency calculation.

(2) For multivariate analyses, four Cox models including different prognostic factors were used. The first model included 4 clinical parameters (International Prognostic Index, gender, B-symptoms, and tumor size), *MYC*, *BCL2*, and *BCL6* translocation status, and MYC, BCL2, and BCL6 protein expression levels as variables (totally 10 factors) and the results are as shown in the top six rows. The second model included clinical parameters, and status for *MYC*+/*BCL2*+, *MYC*+/*BCL6*+, *BCL2*+/*BCL6*+, MYC^+^/BCL2^+^, MYC^+^/BCL6^+^ and BCL2^+^/BCL6^+^ (totally 10 factors) and the results are shown in the 7th-12th rows. The third models included clinical parameters, and status for “MYC^+^/BCL2^+^/BCL6^−^ and MYC^+^/BCL6^+^/BCL2^−^ (totally 6 factors) and the results are shown in the 13-14 rows. The fourth model included clinical parameters and MYC^+^/BCL2^+^/BCL6^+^ as a factor and the results are shown in the last row.

Using immunohistochemistry 249 (30.2%), 439 (51.7%), and 555 (62.6%) patients had high levels of MYC (≥70%), BCL2 (≥70%), and BCL6 ( > 50%) expression, respectively. MYC expression was similarly distributed between GCB and ABC subtypes (*P* = 0.17) whereas BCL2 and BCL6 expression were significantly more common in the ABC and GCB subtypes, respectively (both *P* < 0.0001), in contrast to the association of their gene translocations with GCB and ABC subtype respectively. MYC^+^/BCL2^+^, MYC^+^/BCL6^+^, and BCL2^+^/BCL6^+^ coexpression were observed in 146 (17.6%), 178 (21.7%), and 282 (33.4%) cases, respectively. Unlike the predominance of MYC^+^BCL2^+^ coexpression in the ABC subtype (*P* = 0.0079), both MYC^+^/BCL6^+^ and BCL2^+^/BCL6^+^ coexpression were equally distributed between the two COO subtypes (*P* = 0.35 and *P* = 0.47, respectively). All MYC protein positive patients were further stratified into three subgroups: MYC^+^/BCL2^+^/BCL6^−^ (MYC^+^/BCL2^+^ coexpression, BCL6^−^), MYC^+^/BCL6^+^/BCL2^−^ (MYC^+^/BCL6^+^ coexpression, BCL2^−^) and MYC^+^/BCL2^+^/BCL6^+^ coexpression. The MYC^+^BCL2^+^BCL6^−^ subgroup was predominantly of ABC subtype (*P* = 0.0019), whereas the MYC^+^BCL6^+^BCL2^−^ subgroup was more commonly of GCB subtype (*P* = 0.011) (Table 1).

### Clinicopathologic features of DLBCL with concurrent rearrangement and coexpression

The clinicopathologic features of patients in the study cohort with or without concurrent gene rearrangements and protein coexpression are listed in Table 2. DLBCL patients with *MYC/BCL2* rearrangements more frequently had large tumors (*P* = 0.02) and a lower complete response rate (*P* = 0.0033), and commonly the tumors were of GCB subtype (*P* < 0.0001). No clinicopathologic features were significantly different between DLBCL patients with concurrent *MYC/BCL6* rearrangements *versus* patients without *MYC/BCL6* concurrent rearrangement, although larger tumor size was of borderline significance (*P* = 0.058).

**Table 2:** Clinical characteristics of patients with concurrent *MYC*, *BCL2* or *BCL6* translocations and MYC, BCL2 or BCL6 protein co-expression

Parameters	*MYC*+*/BCL2*+ (DH)	Non- *MYC*+*/BCL2*+	*P*	*MYC*+*/BCL6*+	Non- *MYC*+*/BCL6*+	*P*	MYC^+^/BCL2^+^ (DP)	Non- MYC^+^/BCL2^+^	*P*	MYC^+^/BCL6^+^	Non- MYC^+^/BCL6^+^	*P*	MYC^+^/BCL2^+^, BCL6^−^	MYC^+^/BCL6^+^, BCL2^−^	*P*
	N (%)	N (%)		N (%)	N (%)		N (%)	N (%)		N (%)	N (%)		N (%)	N (%)	
**Age**															
<60	7 (35)	254 (39.9)	.73	4 (30.8)	253 (38.8)	.51	39 (28.7)	285 (43.2)	**.0016**	61 (36.5)	262 (40.4)	.36	14 (34.1)	36 (50)	.10
≥60	13 (65)	401 (60.1)		9 (69.2)	381 (61.2)		97 (71.3)	374 (56.8)		106 (63.5)	386 (59.6)		27 (65.9)	36 (50)	
**Gender**															
F	6 (30)	266 (39.6)	.35	7 (53.8)	252 (40.4)	.30	61 (44.5)	272 (41.2)	.47	74 (44)	266 (41)	.47	17 (40.5)	30 (41.1)	.95
M	14 (70)	392 (60.4)		6 (46.2)	385 (59.6)		76 (55.5)	388 (58.8)		94 (56)	383 (59)		25 (59.5)	43 (58.9)	
**Stage**															
I-II	5 (27.8)	292 (45.2)	.13	5 (41.7)	278 (45.8)	.81	40 (30.5)	318 (49.9)	**<.0001**	60 (37)	299 (47.7)	**.015**	16 (39)	36 (50)	.26
III-IV	13 (72.2)	345 (54.8)		7 (58.3)	337 (54.2)		91 (69.5)	319 (50.1)		102 (63)	328 (52.3)		25 (61)	36 (50)	
**B-symptoms**															
No	11 (61.1)	372 (61.3)	.98	6 (50)	357 (60.9)	.43	70 (55.6)	393 (63.9)	.078	96 (60.8)	377 (62.6)	.67	22 (53.7)	48 (66.7)	.17
Yes	7 (38.9)	239 (38.7)		6 (50)	225 (39.1)		56 (44.4)	222 (36.1)		62 (39.2)	225 (37.4)		19 (46.3)	24 (33.3)	
**LDH**															
Normal	5 (27.8)	244 (39.5)	.25	4 (30.8)	226 (41.1)	.52	44 (35.2)	243 (40.5)	.27	59 (37.6)	239 (40.6)	.48	12 (31.6)	27 (38.6)	.47
Elevated	13 (72.2)	349 (60.5)		9 (69.2)	346 (58.9)		81 (64.8)	357 (59.5)		98 (62.4)	349 (59.4)		26 (68.4)	43 (61.4)	
**# of extranodal sites**															
0-1	12 (63.2)	481 (77.4)	.16	7 (63.6)	468 (77)	.28	88 (67.7)	501 (79.8)	**.0026**	113 (70.2)	490 (79.5)	**.011**	28 (70)	53 (74.6)	.60
≥2	7 (36.8)	144 (22.6)		4 (36.4)	137 (23)		42 (32.3)	127 (20.2)		48 (29.8)	126 (20.5)		12 (30)	18 (25.4)	
**ECOG**															
0-1	14 (77.8)	471 (82.5)	.62	7 (63.6)	456 (82.3)	.11	93 (75)	486 (84.4)	**.012**	119 (76.8)	470 (83.3)	.06	29 (76.3)	56 (81.2)	.55
≥2	4 (22.2)	101 (17.5)		4 (36.4)	97 (17.7)		31 (25)	90 (15.6)		36 (23.2)	94 (16.7)		9 (23.7)	13 (18.8)	
**Size of largest tumor**															
<5cm	4 (25)	273 (54)	**.02**	2 (22.2)	259 (54.4)	.058	50 (45)	288 (56.4)	**.03**	67 (48.2)	268 (56.1)	.10	11 (31.4)	28 (45.2)	.19
≥5cm	12 (75)	229 (46)		7 (77.8)	221 (45.6)		61 (55)	223 (43.6)		72 (51.8)	210 (43.9)		24 (68.6)	34 (54.8)	
**IPI score**															
0-2	8 (44.4)	377 (59.8)	.17	6 (50)	355 (60.5)	.50	56 (42.7)	401 (65.1)	**<.0001**	81 (49.7)	381 (63.2)	**.0018**	19 (47.5)	44 (61.1)	.16
3-5	10 (55.6)	246 (40.2)		6 (50)	239 (39.5)		75 (57.3)	215 (34.9)		82 (50.3)	222 (36.8)		21 (52.5)	28 (38.9)	
**Therapy response**															
CR	10 (55)	487 (75.3)	**.026**	8 (66.7)	464 (76.3)	.48	79 (48.2)	501 (78.8)	**<.0001**	108 (65.9)	484 (77.4)	**.0023**	25 (61)	55 (77.5)	.06
PR	3	80		2	80		59	70		31	70		9	8	
SD	2	27		1	24		9	23		7	30		4	2	
PD	4	44		1	47		17	42		18	41		3	6	
**Cell-of-origin**															
GCB	19 (95)	340 (52.2)	**<.0001**	9 (64.3)	347 (49.4)	.43	62 (42.5)	372 (54.9)	**.0079**	97 (54.5)	337 (50.4)	.35	13 (30.2)	49 (65.3)	**.0003**
ABC	1 (5)	348 (47.8)		5 (35.7)	318 (50.6)		84 (57.5)	306 (45.1)		81 (45.5)	332 (49.6)		30 (69.8)	26 (34.7)	
**Ki-67 index**															
<70%	5 (45.5)	137 (32.5)	.52	3 (37.5)	125 (33.1)	.72	19 (19.4)	170 (39.2)	**.0002**	29 (23.8)	159 (39.6)	**.0017**	6 (19.4)	16 (28.6)	.44
≥70%	6 (54.5)	277 (67.5)		5 (62.5)	260 (66.9)		79 (80.6)	264 (60.8)		93 (76.2)	243 (60.4)		25 (80.6)	40 (71.4)	
***TP53***mutation															
WT	8 (72.7)	301 (78.8)	.71	7 (100)	276 (78.2)	.35	62 (71.3)	311 (78.9)	.12	81 (75.7)	284 (78.0)	.60	20 (76.9)	40 (83.3)	.54
MUT	3 (27.3)	81 (21.2)		0 (0)	77 (21.8)		25 (28.7)	83 (21.1)		26 (24.3)	80 (22.0)		6 (23.1)	8 (16.7)	

**Abbreviations**: DH, double-hit; DP, double-positive; LDH, lactate dehydrogenase; ECOG, Eastern Cooperative Oncology Group performance status; IPI, International Prognostic Index; CR, complete remission; PR, partial response; SD, stable disease; PD, progressive disease; GCB, germinal center B-cell like; ABC, activated B-cell like; WT, wild type; MUT, mutant. For therapy response, we calculated *P* values as CR vs other responses. Some clinical features of certain cases were not available.

Patients with MYC^+^/BCL2^+^ coexpression were more often of older age (*P* = 0.0016) and more often had advanced disease stage (*P* < 0.0001), extranodal involvement (*P* = 0.0026), large tumor size (*P* = 0.03), International Prognostic Index score > 2 (*P* < 0.0001), low complete response rate (*P* = 0.0071), and high Ki-67 (*P* = 0.0002), and the lymphoma was more often of ABC subtype (*P* = 0.0079). Patients with MYC^+^/BCL6^+^ coexpression were associated with advanced disease stage (*P* = 0.015), extranodal sites (*P* = 0.011), low complete response rate (*P* = 0.0023) and high Ki-67 index (*P* = 0.0017). When MYC^+^/BCL2^+^/BCL6^−^ and MYC^+^/BCL6^+^/BCL2^−^ subgroups were isolated from the MYC^+^/BCL2^+^ and MYC^+^/BCL6^+^ patients respectively, MYC^+^/BCL2^+^/BCL6^−^ tumors were more often of ABC subtype, whereas MYC^+^/BCL6^+^/BCL2^−^ tumors were more commonly of GCB subtype (*P* = 0.0003) (Table 2).

### Prognostic impact of concurrent rearrangements of *MYC*, *BCL2*, and *BCL6* in DLBCL

**Figure 1:**
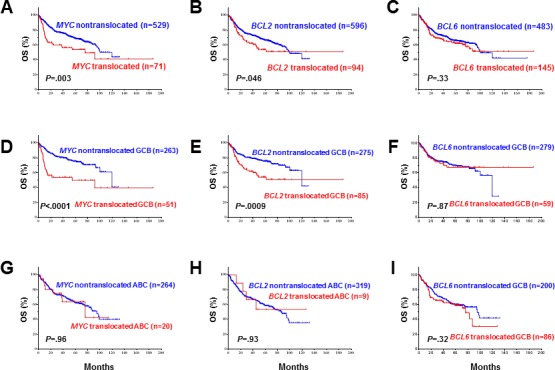
Univariate analysis for patients with DLBCL with *MYC*, *BCL2*, and *BCL6* rearrangemnts in the overall-, GCB, and ABC groups **A.**-**B.**, **D.**-**E**, **G.-H.** *MYC* and *BCL2* rearrangements correlated with significantly poorer overall survival in overall and GCB- but not ABC-DLBCL. **C., F., I.** *BCL6* translocation did not correlate with poorer overall survival.

We first assessed the prognostic impact of rearrangements of *MYC*, *BCL2*, and *BCL6* in DLBCL (Figure 1 and Supplementary Figure S1). Both *MYC* and *BCL2* rearrangements correlated to a poorer survival in the whole cohort (*P* = 0.003 for *MYC*, Figure 1A; *P* = 0.046 for *BCL2*, Figure 1B) and in the GCB subtype (*P* < 0.0001 for *MYC*, Figure 1D; *P* < 0.0009 for *BCL2*, Figure 1E) but not in the ABC subtype (Figure 1G–1H). *BCL6* translocation, by contrast, did not correlate with poorer survival for either the whole cohort (*P* = 0.33, Figure 1C) or in the GCB (*P* = 0.87, Figure 1F) and ABC (*P* = 0.32, Figure 1I) subtypes.

**Figure 2:**
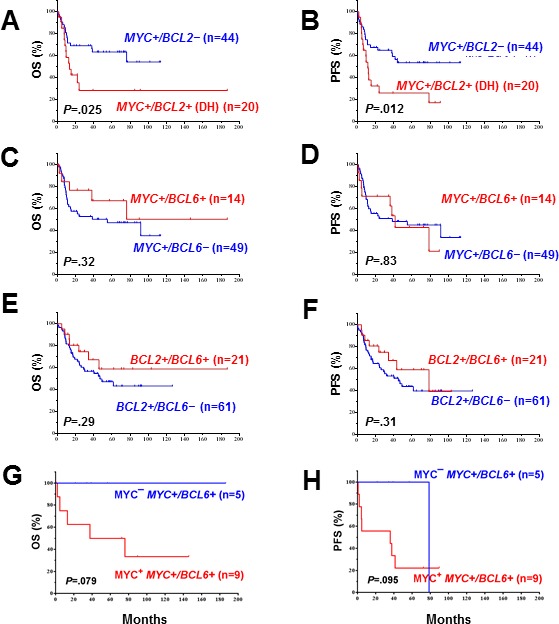
**A.-B.** The prognostic significance of *MYC* rearrangements in DLBCL depends on *BCL2* rearrangement. **C.-D.** *BCL6* rearrangement had no additive effect to *MYC* rearrangements. **E.-F.** *BCL6* translocation had no additive effect to BCL2 rearrangements. **G.-H.** MYC expression levels appeared to impact the survival of *MYC*+/*BCL6+* rearranged DLBCL with marginal *P* values probably due to the small case numbers.

We then assessed the prognostic impact of concurrent rearrangements in DLBCL. Patients with *MYC/BCL2* translocations had a worse survival than patients with *MYC* rearrangement alone (overall survival [OS]: *P* = 0.025; PFS: *P* = 0.012, Figure 2A–2B). However, no difference was observed in OS and PFS between DLBCL patients with *MYC/BCL6* rearrangement *versus* DLBCL patients with only *MYC* rearrangement (Figure 2C–2D). There was also no difference in OS and PFS between patients with DLBCL with *BCL2/BCL6* rearrangement *versus* DLBCL with only *BCL2* rearrangement (Figure 2E–2F). Notably the survival of patients with *MYC/BCL6* rearrangement appeared to be affected by MYC expression levels (Figure 2G–2H).

**Figure 3:**
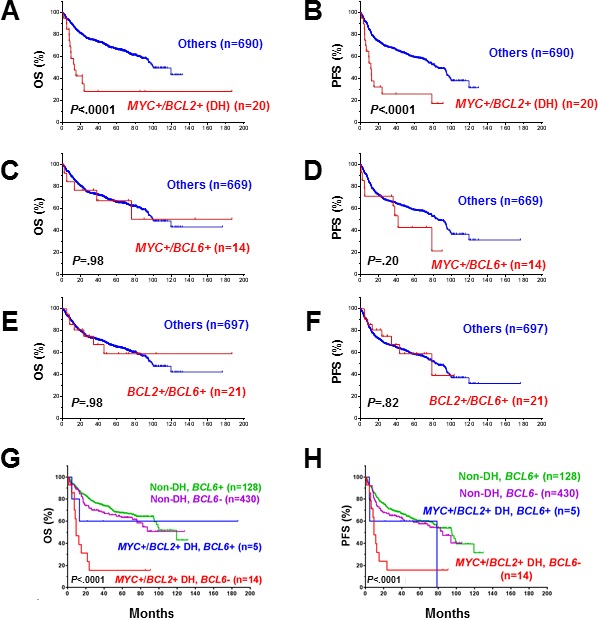
**A.-B.** Concurrent *MYC*/*BCL2* rearrangements correlated with significant poorer overall survival. **C.-D.** Concurrent *MYC*+/*BCL6*+ rearrangements did not correlate with poorer overall survival. **E.-F.** Concurrent *BCL2*+/*BCL6*+ rearrangements did not correlate with poorer overall survival. **G.-H.** BCL6 attenuated the adverse prognostic impact of *MYC*+/*BCL2*+ double-hit lymphoma.

The prognostic differences between patients with concurrent rearrangements of *MYC/BCL2*, *MYC/BCL6*, *BCL2/BCL6 versus* the remaining DLBCL patients are shown in Figure 3. Only concurrent *MYC/BCL2* rearrangements correlated with significantly poorer survival (Figure 3A–3B). Additional *BCL6* translocation (triple-hit, *n* = 5, 26% of 19 *MYC*+*/BCL2*+ cases with *BCL6* translocation status available) had no synergistic effect with concurrent *MYC/BCL2* rearrangements and, on the contrary, attenuated the adverse impact of concurrent *MYC/BCL2* rearrangements (Figure 3G–3H).

### Prognostic impact of coexpression of MYC, BCL2, and BCL6 in DLBCL

**Figure 4:**
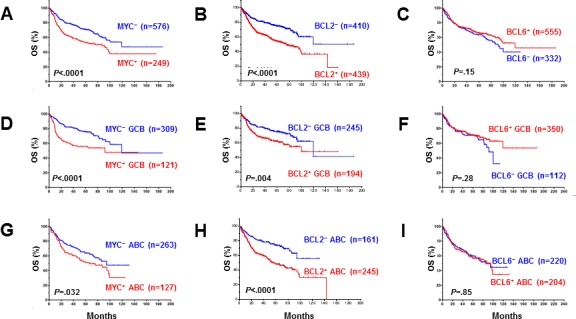
Univariate analysis for DLBCL patients with MYC, BCL2 and BCL6 protein expression in the overall-, GCB, and ABC-DLBCL **A.-B., D.-E., G.-H.** MYC and BCL2 protein expression correlated with significantly poorer overall survival in overall, GCB- and ABC-DLBCL. **C., F., I.** BCL6 overexpression did not correlate with poor survival.

The prognostic impact of protein expression of MYC, BCL2, and BCL6 is shown in Figure 4 and Supplementary Figure S2. BCL6 expression in DLBCL did not correlate with poorer patient survival, either in the whole group or in COO subtypes (Figure 4C, 4F, and 4I). In contrast, MYC^+^ or BCL2^+^ expression in DLBCL correlated with significantly poorer survival for the overall patient cohort (*P* < 0.0001, Figure 4A–4B) and for patients with GCB (MYC^+^: *P* < 0.0001, Figure 4D; BCL2^+^: *P* = 0.004, Figure 4E) and ABC subtypes of DLBCL (MYC^+^: *P* = 0.032, Figure 4G; BCL2^+^: *P* < 0.0001, Figure 4H).

**Figure 5:**
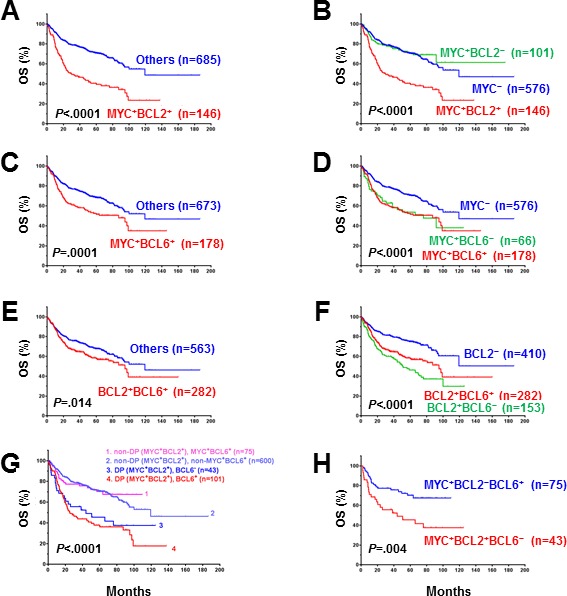
**A., C., E.** Patients with DLBCL and MYC/BCL2, BCL6/MYC or BCL2/BCL6 co-expression had significantly poorer overall survival in the DBLCL cohort. **B.** BCL2 overexpression had a synergetic effect with MYC overexpression and the adverse prognostic impact of MYC depended on BCL2 overexpression. **D.** BCL6 expression had no synergetic effect with MYC expression. **F.** BCL6 expression appeared to attenuate the adverse prognostic impact of BCL2 overexpression. **G.** The poorer overall survival of MYC^+^BCL6^+^ patients was due to the poor survival of MYC^+^BCL2^+^ patients. **H.** Isolated MYC^+^BCL6^+^ *versus* MYC^+^BCL2^+^ double-positive DLBCL had significantly better patient survival.

Our results further showed that MYC^+^/BCL2^+^ (*P* < 0.0001, Figure 5A, Supplementary Figure S3A), MYC^+^/BCL6^+^ (OS: *P* = 0.0001, Figure 5C; PFS: *P* = 0.0002, Supplementary Figure S3C), and BCL2^+^/BCL6^+^ (OS: *P* = 0.014, Figure 5E; PFS: *P* = 0.033, Supplementary Figure S3E) coexpression correlated with significantly poorer survival in the overall cohort. The inferior survival of patients MYC^+^/BCL2^+^ and MYC^+^/BCL6^+^ DLBCL compared with all other DLBCL patients was significant for both the GCB and ABC subtypes, whereas BCL2^+^/BCL6^+^ only correlated with poorer OS for patients with ABC-DLBCL (Supplementary Figure S4). MYC expression showed dependence and synergy only with BCL2 expression (Figure 5B, Supplementary Figure S3B); BCL6 expression had no additive adverse impact in patients with MYC^+^, BCL2^+^ or MYC^+^/BCL2^+^ DLBCL (Figure 5D, 5F–5G, Supplementary Figure S3D, S3F-S3G). Patients with MYC^+^/BCL2^+^ DLBCL but not MYC^+^/BCL6^+^ DLBCL had poorer survival (Figure 5G, Supplementary Figure S3G). The poor prognosis of patients with MYC^+^/BCL6^+^ DLBCL was attributable to the poorer survival of MYC^+^/BCL2^+^ DLBCL patients.

We further evaluated the survival of patients with isolated MYC^+^/BCL2^+^/BCL6^−^ (i.e., BCL6^−^ DPL) *versus* isolated MYC^+^/BCL6^+^/BCL2^−^ (i.e., BCL2^−^ DPL) DLBCL. Patients with isolated MYC^+^/BCL6^+^ DLBCL had favorable survival compared with isolated MYC^+^/BCL2^+^ DLBCL patients (OS: *P* = 0.004, Figure 5H; PFS: *P* < 0.0001, Supplementary Figure S3H).

### Gene expression signatures of concurrent *MYC*, *BCL2*, and *BCL6* rearrangements and isolated MYC^+^BCL2^+^ and MYC^+^BCL6^+^ coexpression

To better understand the molecular mechanisms of the effects on prognosis, we compared the GEP of patients with concurrent *MYC/BCL2* or *MYC/BCL6* rearrangements with the remaining patients. Only *MYC/BCL2* rearranged DLBCL showed a distinctive GEP signature, whereas concurrent *MYC/BCL6* rearranged DLBCL did not show DEGs compared with *MYC* rearranged DLBCL or the remaining DLBCL patients. The GEP signature of concurrent *MYC/BCL2* rearranged DLBCL included 24 upregulated genes and 14 downregulated genes at the false discovery rate threshold of 0.01 (Table. 3A, Figure 5A). These genes were involved in signaling (upregulation of *BMP3*, *SWAP70*, and *CELSR1*, and downregulation of *PLA2G7* and *DOCK10*), cell proliferation (upregulated *STRBP* and *MUC4*), metabolism (eight upregulated genes), apoptosis (*TMEM49*, *CFLAR*, *CARD16* and *CASP1*), transcription factors and genes related to cell adhesion, extracellular matrix, and migration (*MYO3B*, *SLAMF7*, *SILEC10/12*, *TPM4,* and *SRGN*).

**Table 3:** GEP signatures of *MYC*/*BCL2* double-hit lymphoma, isolated MYC^+^BCL2^+^ (i.e., BCL6^−^ DPL) and MYC^+^BCL2^−^BCL6^+^ (i.e., BCL2^−^ DPL)

**A. *MYC*+/*BCL2* + double-hit *vs* others (false discovery rate < .01)**		
**Functional categories**	**Upregulated genes**	**Downregulated genes**
Signaling	*BMP3*, *SWAP70*, *CELSR1*	*PLA2G7*, *DOCK10*, *RNF149*
Cell proliferation and growth	*STRBP*, *MUC4*	
Transcriptional regulation	*ZNF280D*, *DIP2C*	*PRDM1*
Metabolism	*RIMKLB*, *UGT8*, *PLA2G12A*, *SLC25A27*, *CYP39A1*, *C7orf10*, *ENPP3*, *HPGD*	
Cell death		*TMEM49*, *CFLAR*, *CARD16 /CASP1*
Immune response	*NCR3LG1*	
Cell adhesion, extracellular matrix, migration	*MYO3B*	*SLAMF7*, *SIGLEC10/12*, *TPM4*, *SRGN*
microRNA		*MIR21*
Others	*TPD52*, *C4orf34*, *FLJ41455*, *XKR6*, *FAM53B*, *TMEM156*, *CEACAM7*	*FLCN*
**B. MYC^+^BCL2^+^BCL6^−^*vs* MYC^+^BCL2^−^BCL6^+^ (false discovery rate < .05)**		
	**Upregulated in MYC^+^BCL2^+^BCL6^−^**	**Upregulated in MYC^+^BCL2^−^BCL6^+^**
BCR signaling, receptors, antigen, modulators, transducers	*CCR7*, *CYSLTR1*, *PRSS21*	*STAP1*, *SORL1*, *RFTN1*, *GNA13*, *SWAP70*, *ANKRD13A*, *PIK3CG*
Proliferation, cell cycle, gene expression DNA replication	*SUB1*, *ZBTB32*, *LIMD1*, *C16orf53*	*BCL6*, *MYBL1*, *NEK6*, *BRWD1*, *SFRS15*, *HMGN1*, *TMPO*
Apoptosis	*BCL2*, *CASP10*	*DNAJC10*
DNA repair		*MSH6*
Metabolism	*ALDH4A1*	*BPNT1*
Cell shape, cytoskeleton, microtubes, migration	*BBIP10*	*MARCKSL1*, *VNN2*, *OSBPL3*, *ACTR2*
Unknown function	*LOC100289094*	*C17orf99*, *KIAA0746*, *ZNF608*

Abbreviations: DH, double-hit; DP, double-positive.

**Figure 6:**
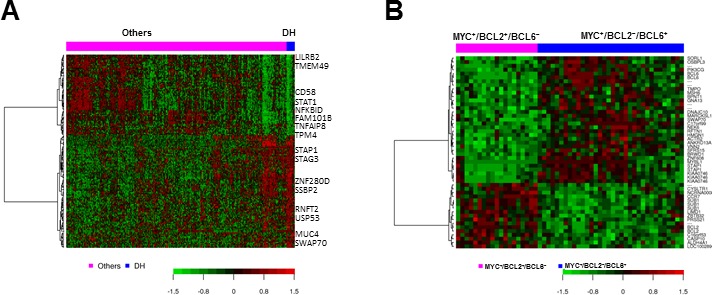
Gene expression signature for *MYC/BCL2* double-hit DLBCL (A) and comparison of MYC^+^BCL2^+^ BCL6^−^ versus MYC^+^BCL2^−^BCL6^+^ translocation in DLBCL (B)

Comparison of the GEP of MYC^+^ BCL6^+^ *versus* MYC^+^BCL2^+^ DLBCL did not show significant DEGs. We further compared the GEP of isolated MYC^+^BCL2^−^BCL6^+^ DLBCL *versus* isolated MYC^+^BCL2^+^BCL6^−^ DLBCL, which resulted in 36 DEGs at the false discovery rate threshold of 0.05 (Table 3B, Figure 6B). Upregulated genes in MYC^+^BCL2^−^BCL6^+^ compared with MYC^+^BCL2^+^BCL6^−^ included *BCL6*, a proliferation signature (*MYBL1*, *NEK6*, *BRWD1* [bromodomain and WD repeat domain containing 1], *SFRS15*, *HMGN1*, and *TMPO*), *MSH6* involved in DNA repair, *DNAJC10* which promotes apoptotic in response to endoplasmic reticulum stress, various signaling genes including *PIK3CG* (PI3K catalytic subunit gamma), *STAP1* (B-cell receptor signaling) *SORL1*, *RFTN1*, *GNA13*, *SWAP70*, and *ANKRD13A*, and genes involved in actin cytoskeleton regulation and migration (*MARCKSL1*, *VNN2*, *OSBPL3*, *ACTR2* and *etc.*). Comparably, upregulated genes in MYC^+^BCL2^+^BCL6^−^ included signatures of apoptosis (antiapoptotic *BCL2*, and paradoxically proapoptotic, *CASP10*), glutamine metabolism (*ALDH4A1*), indicating that abnormal dysregulation of apoptotic and proliferation pathways are critical in patients with MYC^+^BCL6^+^BCL2^−^. In contrast, such signaling and molecular defects were not seen in patients with MYC^+^BCL6^−^BCL2^+^. The observations provide molecular basis for the difference of outcome and survival between these two groups of DLBCL patients.

## DISCUSSION

In the literature B-cell lymphomas with concurrent *MYC/BCL2* or *MYC*/*BCL6* rearrangements are grouped together as double-hit B-cell lymphomas [1, 2]. Patients with *MYC/BCL2* DHL have responded poorly to all traditional chemotherapy regimens and have extremely poor outcomes [1, 10, 11]. However, most of what we know about double-hit B-cell lymphoma is derived from studies of the most common form, *MYC/BCL2* DHL. In contrast, very little has been published on DLBCL patients with concurrent *MYC/BCL6* rearrangements. The findings in this study for a group of DLBCL patients treated with R-CHOP suggest that patients with concurrent *MYC/BCL6* rearrangements do not have a poorer prognosis and that grouping these tumors with other forms of DHL could lead to inappropriate therapy.

In this large cohort of 898 cases of *de novo* DLBCL, rearrangements of *MYC, BCL2,* and *BCL6* were found in 11.8%, 13.6% and 23.1% of cases, respectively. *BCL6* rearrangements were more frequently observed in the ABC subtype whereas *MYC* and *BCL2* rearrangements were more frequently observed in the GCB subtype. Concurrent *MYC*/*BCL6* and *MYC*/*BCL2* rearrangements were observed in 2.0% and 2.8% of DLBCL cases, respectively. *MYC*/*BCL2*/*BCL6* triple-hit was observed in 38% of *MYC*/*BCL6* and 26% of *MYC*/*BCL2* double-hit cases. The frequencies of *MYC*, *BCL2*, and *BCL6* rearrangement and concurrent *MYC/BCL2* rearrangements are similar to those reported in DLBCL in earlier studies [10, 11, 31–34].

In this study, DLBCL patients with concurrent *MYC/BCL6* rearrangements did not have a poorer prognosis. The notion that concurrent *MYC/BCL6* rearrangements in DLBCL is not an indication of aggressive lymphoma was further substantiated by the lack of a distinctive GEP signature for *MYC/BCL6* rearranged DLBCL although BCL6 was overexpressed in almost all *MYC/BCL6* rearranged patients except one case with an IHC of 30%. These phenomena might be explained by a previous finding that up to 43% *BCL6* translocations involved non-*IG* loci showing complex gene expression patterns [35], limited case numbers, and 36% of *MYC*/*BCL6* cases showing low MYC expression levels (Figure 2G–2H). In contrast, *MYC/BCL2* rearranged DLBCL correlated with significantly poorer survival, and was associated with a distinctive GEP signature suggesting increased proliferation, growth and metabolism and decreased apoptosis pathway (our results showed downregulation of both pro- and anti-apoptotic genes).

In the MD Anderson Cancer Center experience with 52 DHL patients tested for *BCL6*, 24 patients with *BCL6* gene abnormality (translocation or amplification, *n* = 15 and *n* = 9 respectively. Among them, 14 patients had *MYC*/*BCL2*/*BCL6* triple-hit) showed slightly better survival than other patients with DHL (hazard ratio: 0.59, 95% confidence interval: 0.21-1.69, *P* = 0.33) [36]. Ueda *et al.* presented a case report consistent with our results, in which a person having DLBCL with concurrent *MYC*, *BCL2*, and *BCL6* rearrangements achieved complete remission after chemoradiotherapy for two years [37]. A recent study reported the largest DHL series including 41 cases with *BCL6* rearrangement, in which patients' OS was not significantly affected by whether the DHL was *MYC*/*BCL2* or *MYC*/*BCL6* (*P* = 0.537). However, 25 (58.5%) of the 41 *MYC*/*BCL6* patients also had *BCL2* rearrangement (triple-hit) [38]. In contrast, in another study, *MYC*/*BCL6* (*n* = 13) showed significantly worse survival than *MYC*/*BCL2* DHL (*n* = 20) after exclusion of triple-hit lymphoma [39]. These *MYC*/*BCL6* DHL showed a trend toward higher *MYC* mRNA expression and a distinct gene expression profile compared to *MYC*/*BCL2* DHL. In a smaller study reported by Pillai *et al*. B-cell lymphoma with concurrent *MYC/BCL6* rearrangements was associated with aggressive clinical course and poor survival [40]. A possible explanation for the discrepancy is patient selection. In the study by Pillai *et al*, patients with BL, BL-like lymphoma, and primary effusion lymphoma were also included. Moreover, the median age of their patients was 83 years and only one of six patients with adequate information received chemotherapy in combination with rituximab [40]. In this study, all patients met morphologic and immunophenotypic criteria for DLBCL with a median patient age of 64 years, which is comparable to that of patients with DLBCL without concurrent *MYC/BCL6* rearrangements, and all patients were treated with standard R-CHOP therapy.

Recently, the concept of *MYC/BCL2* rearranged DHL has been extended to MYC and BCL2 protein coexpression [11, 22, 23]. These studies showed that patients with DLBCL with MYC/BCL2 double-positive (by immunohistochemistry) also have a dismal prognosis, regardless of the status of *MYC* or *BCL2* rearrangement [11, 22, 23]. In this study, DLBCL patients with MYC/BCL6 coexpression showed a significantly poorer survival than DLBCL patients without MYC/BCL6 coexpression. However, this prognostic effect was significant only in the presence of DLBCL with MYC/BCL2 coexpression. The isolated MYC^+^BCL2^−^BCL6^+^ (from all MYC^+^BCL6^+^) subgroup had significantly better patient survival compared with the MYC^+^BCL2^+^BCL6^−^ (from all MYC^+^BCL2^+^) subgroup. Previous studies also suggested that BCL6 expression is associated with favorable survival in patients with DLBCL [24, 30, 41, 42].

There is a biologic basis that may explain the lack of significantly adverse prognostic impact of recurrent *MYC/BCL6* rearrangements and MYC/BCL6 coexpression. BCL6 represses *CCND2* and *MYC* [27–29, 43]; we have recently shown that MYC expression levels significantly impact the prognosis of *MYC* rearranged DLBCL [44], and our multivariate analysis suggested BCL6 expression correlated with favorable survival (*P* = 0.048 for OS and *P* = 0.016 for PFS, Table 1), therefore the adverse prognostic impact of MYC might have been diminished by high BCL6 expression in these cases. In addition to the potential role of BCL6, the GEP signature of MYC^+^BCL2^−^BCL6^+^ DLBCL suggested DNA repair and proapoptosis, in contrast with the upregulation of antiapoptotic *BCL2* in MYC^+^BCL2^+^BCL6^−^ DLBCL. Interestingly, *MYBL1* and *LIMD1* were also significantly upregulated in our MYC^+^BCL2^−^BCL6^+^ (associated with GCB) and MYC^+^BCL2^+^BCL6^−^ (associated with ABC) DLBCL subgroup respectively, which is in consistent with the correlations between a novel two-gene expression index, “LIMD1-MYBL1 Index”, and GCB/ABC subtypes and clinical outcome [45, 46].

In summary, DLBCL patients with concurrent *MYC/BCL6* rearrangements are not necessarily associated with an inferior prognosis when treated with R-CHOP therapy, unlike DLBCL patients with concurrent *MYC/BCL2* rearrangements, probably due to different pathogenesis and MYC expression levels. In addition, DLBCL patients with MYC/BCL6 coexpression did have an inferior prognosis, but only in the presence of MYC/BCL2 coexpression, and therefore MYC/BCL6 coexpression seems to be of less prognostic importance [46]. These data support the notion that DLBCL with concurrent *MYC/BCL6* rearrangements and DLBCL with *MYC/BCL2* DHL are not equivalent prognostically. These results suggest that the concept of double-hit lymphoma needs to be refined. The grouping of cases of DLBCL with concurrent *MYC/BCL6* rearrangements with cases of DLBCL with *MYC/BCL2* rearrangements may lead to over treatment of *MYC*/*BCL6* rearranged DLBCL patients.

## PATIENTS, MATERIALS AND METHODS

### Patients

A cohort of 898 patients with *de novo* DLBCL treated with standard rituximab, cyclophosphamide, doxorubicin, vincristine and prednisolone (R-CHOP) therapy was collected as part of The International DLBCL Rituximab-CHOP Consortium Program Study [33, 46]. All patients were diagnosed according to the World Health Organization (WHO) classification system and received treatment between 1998 and 2010. Cases were excluded if patients had a history of low-grade B-cell lymphoma; human immunodeficiency virus infection; or primary mediastinal, cutaneous B cell lymphoma, or central nervous system DLBCL. This study was approved by the Institutional Review Boards of each participating center, and the comprehensive collaborative study was approved by the Institutional Review Board at The University of Texas MD Anderson Cancer Center.

### Tissue microarray, immunohistochemistry and fluorescence in situ hybridization

Construction of tissue microarrays, IHC staining procedures on tissue microarray sections, and scoring criteria for MYC, BCL2 and BCL6 have been described previously [23, 44, 47]. MYC (clone Y69; Epitomics, Burlingame, CA) and BCL6 (clone LN22; Leica Microsystems, Buffalo Grove, IL) expression showed a distinct nuclear pattern and BCL2 (clone 124; DAKO, Denmark) expression exhibited a cytoplasmic pattern. Cutoffs for MYC, BCL2 and BCL6 overexpression are determined as ≥70%, ≥70%, and > 50% respectively based on survival analysis as described previously, and cutoffs and positivity rates reported by other study groups.

Interphase fluorescence *in situ* hybridization (FISH) analysis was performed using formalin-fixed, paraffin-embedded tissue sections and BCL2 and BCL6 dual-color, break-apart probes (Vysis), IGH/MYC/CEP8 tricolor dual-fusion probes (Vysis) and a locus specific MYC dual-color break-apart probe (Vysis) as described previously [47].

### Gene expression profiling and COO classification

Gene expression profiling (GEP) was performed on formalin-fixed, paraffin-embedded tissue samples using Affymetrix GeneChip HG-U133 Plus Version 2.0 (Affymetrix, Santa Clara, CA) as described in an earlier study [47]. The CEL files are deposited in the National Center for Biotechnology Information Gene Expression Omnibus repository (GSE#31312).^61^ The microarray data were quantified and normalized by the frozen robust multiarray analysis (RMA) algorithm [48]. Differential expression gene (DEG) analysis was performed using multiple *t*-tests [31, 47]. Cell-of-origin classification into either GCB or ABC subtypes was achieved by using either GEP (*n* = 497) or IHC methods (*n* = 401) according to the Visco-Young (first selection) and Choi (the second selection) algorithms [49–53].

### Statistical analysis

Clinical and laboratory features of DLBCL patients at the time of presentation according to different subgroups were compared using the chi-squared test and the Spearman rank correlation test. Overall survival (OS) was calculated from the date of diagnosis to the date of last follow-up or death. Progression-free survival (PFS) was calculated from the date of diagnosis to the time of progression or death. Kaplan-Meier survival curves were used to estimate OS and PFS rates, and the log-rank (Mantel-Cox) test was used to assess differences in survival between groups. Multivariate analysis for survival was performed with IBM statistics SPSS 19 software using the Cox proportional hazards regression model (Chicago, SPSS Inc.). All differences with *P*≤0.05 were considered to be statistically significant.

## SUPPLEMENTARY MATERIAL FIGURES


