# Supplementary material for: Prognostic impact of concurrent MYC and BCL6 rearrangements and expression in de novo diffuse large B-cell lymphoma
Source: Oncotarget. 2015 Nov 12;7(3):2401–16. doi: 10.18632/oncotarget.6262 (PMC4823044; doi:10.18632/oncotarget.6262)
Supplement: Supplementary file 1 [file oncotarget-07-2401-s001.pdf]

Prognostic impact of concurrent *MYC* and *BCL6* rearrangements and expression in *de novo* diffuse large B-cell lymphoma

Supplementary Material

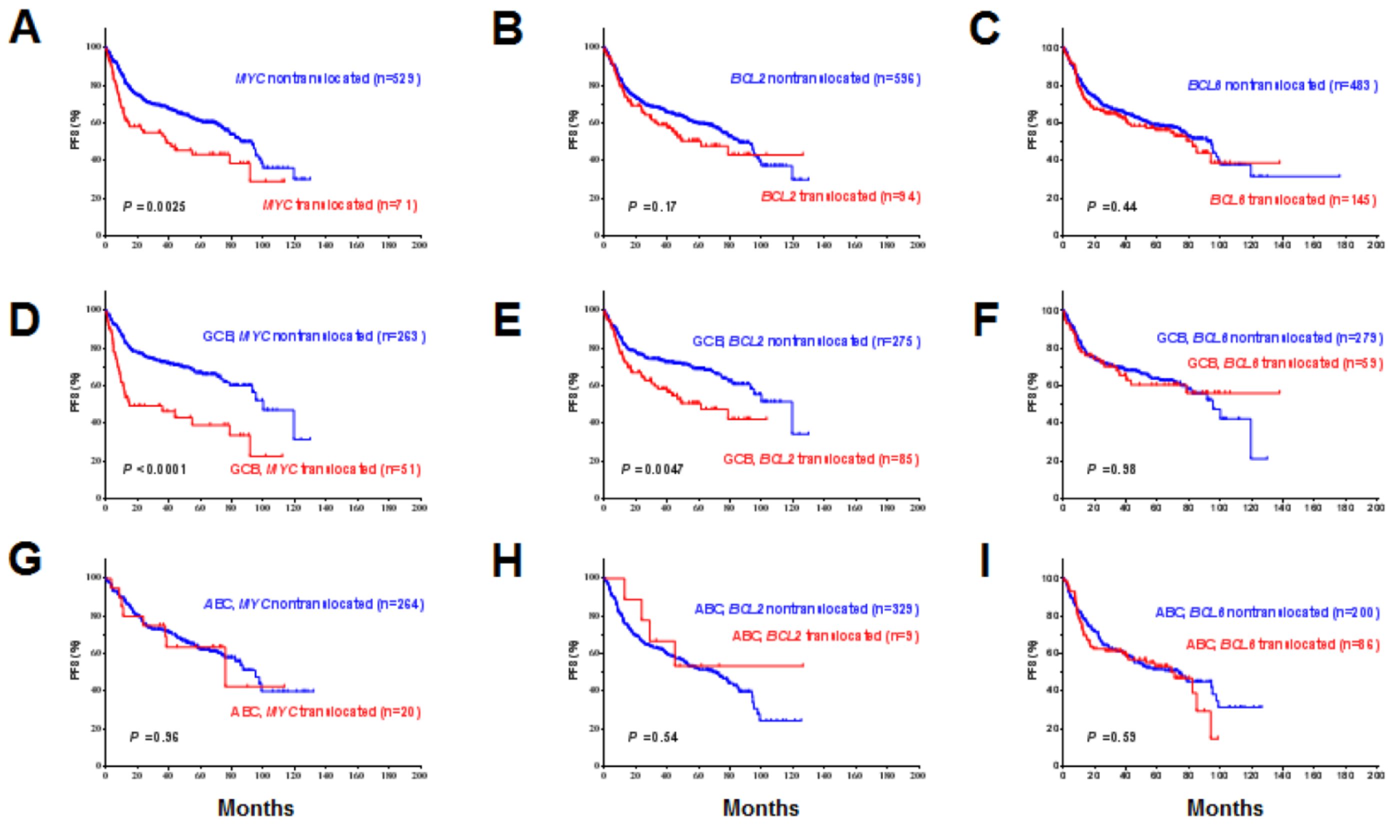

Supplementary Figure S1. Progression-free survival (PFS) curves of the univariate analysis for *MYC*, *BCL2*, and *BCL6* translocations in overall-, GCB, and ABC-DLBCL. **(A-B, D-E, G-H)** *MYC* and *BCL2* translocations correlated with significantly poorer PFS in overall and GCB- but not ABC-DLBCL. **(C,F, I)** *BCL6* translocation did not correlate with poor PFS.

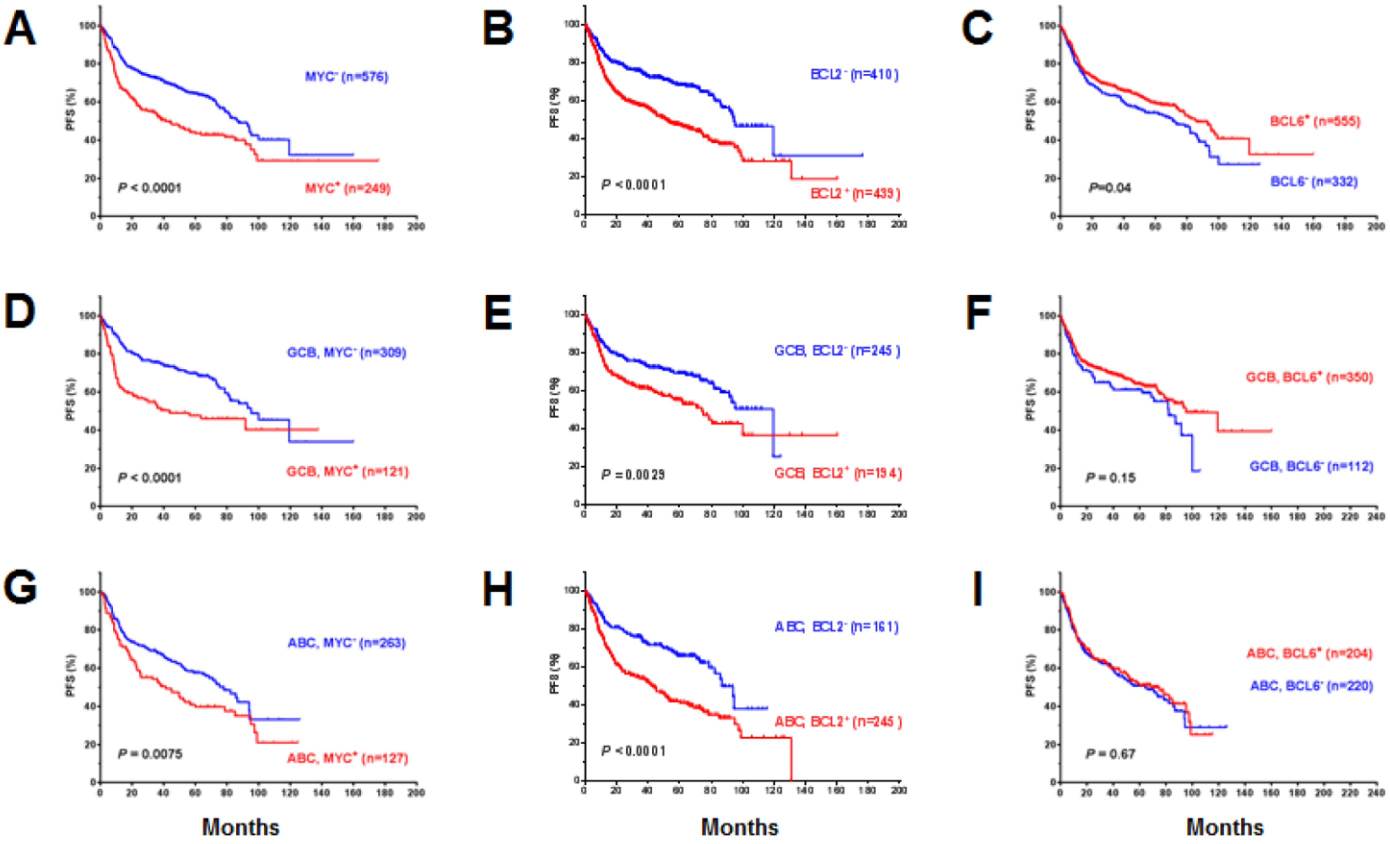

Supplementary Figure S2 Progression-free survival (PFS) curves of the univariate analysis for Myc, Bcl-2 and Bcl-6 protein expression in overall-, GCB, and ABC-DLBCL. **(A-B, D-E, G-H)** MYC and BCL2 protein expression correlated with significantly poorer PFS in overall, GCB- and ABC-DLBCL. **(C,F, I)** BCL6 overexpression did not correlate with poor PFS.

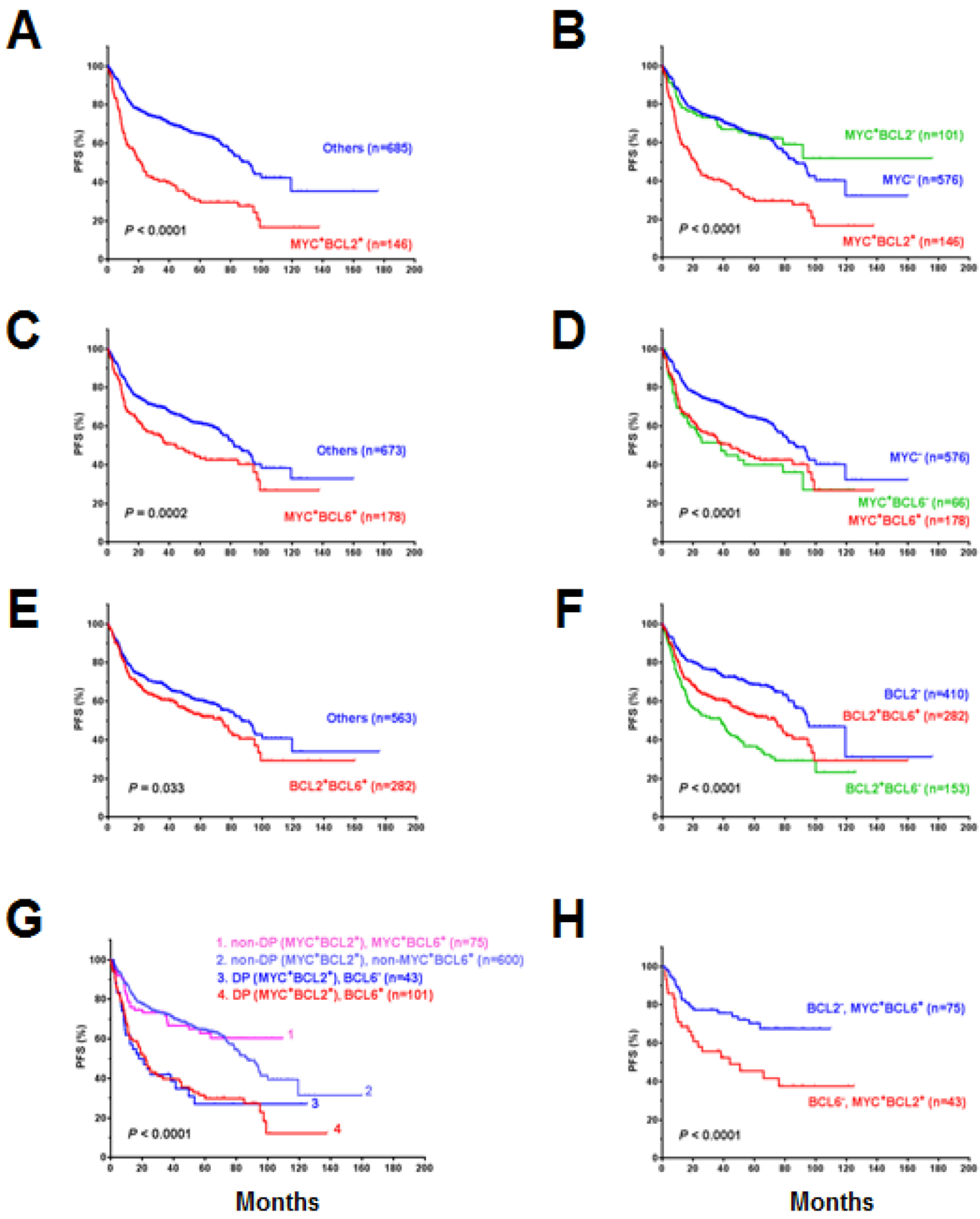

Supplementary Figure S3 (A, C, E) Patients with MYC/BCL2, BCL6/MYC or BCL2/BCL6 co-expression had significantly poorer progression-free survival in DBLCL cohort. **(B)** BCL2 overexpression had synergetic effect with MYC overexpression and the adverse prognostic impact of MYC depended on BCL2 overexpression. **(D)** BCL6 expression had no synergetic effect with MYC expression. **(F)** BCL6 expression appeared to attenuate the adverse prognostic impact of BCL2 overexpression. **(G)** The poor progression-free survival of $MYC^{+}BCL6^{+}$ was due to the poor survival of $MYC^{+}BCL2^{+}$ patients. **(H)** Isolated $MYC^{+}BCL6^{+}$ *versus* $MYC^{+}BCL2^{+}$ double-positive DLBCL had significantly better patient progression-free survival.

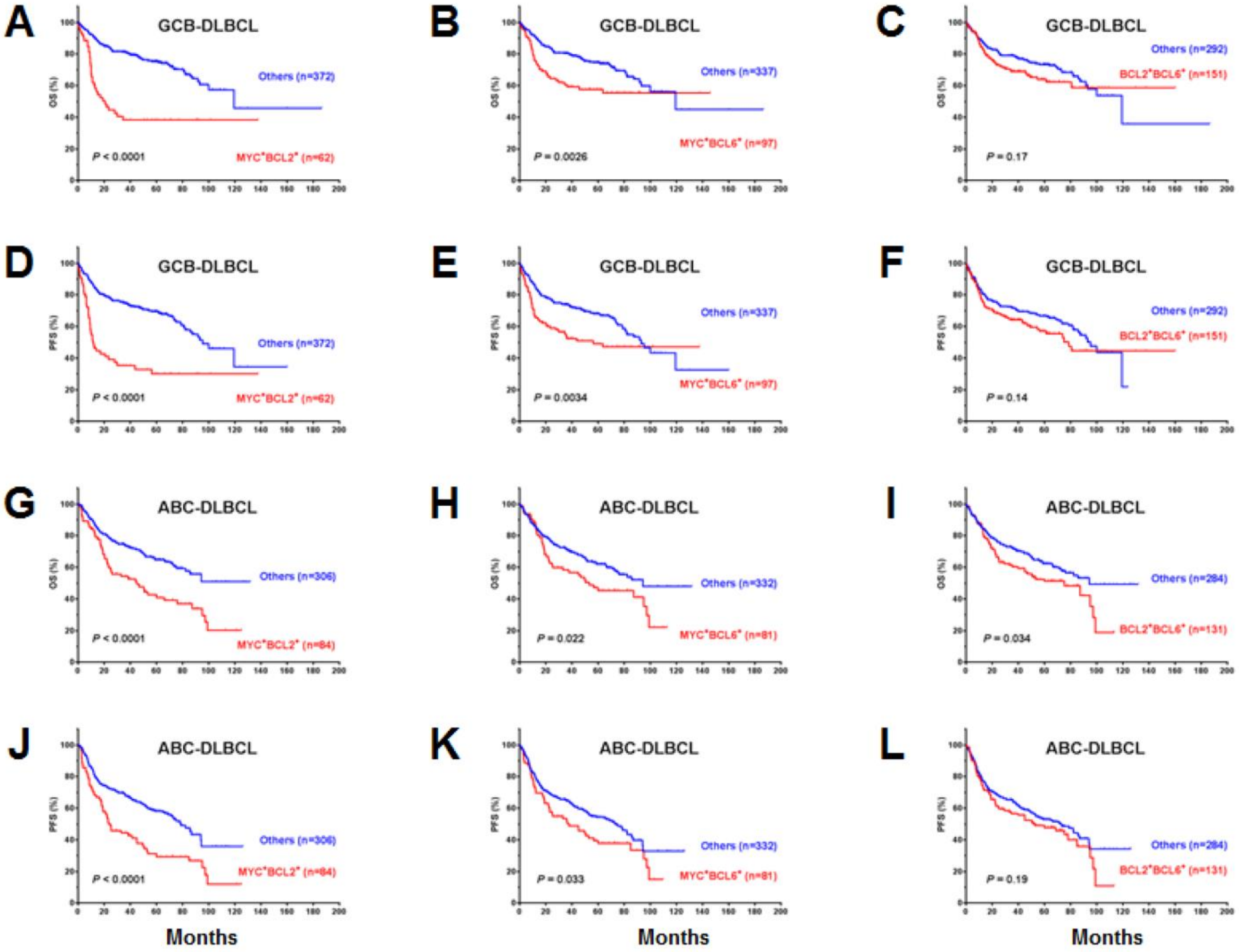

Supplementary Figure S4 Prognostic significance of MYC/BCL2, BCL6/MYC and BCL2/BCL6 co-expression in GCB (Figures A-F) and ABC-DLBCL (Figures G-K) respectively.
